# Remote sensing and model analysis of biomass burning smoke transported across the Atlantic during the 2020 Western US wildfire season

**DOI:** 10.1038/s41598-023-39312-1

**Published:** 2023-09-25

**Authors:** Xavier Ceamanos, Quentin Coopman, Maya George, Jérôme Riedi, Mark Parrington, Cathy Clerbaux

**Affiliations:** 1 CNRM, Météo-France, CNRS, Université de Toulouse, Toulouse, France; 2 Department of Atmospheric and Oceanic Sciences, McGill University, Montreal, QC Canada; 3 LATMOS/IPSL, Sorbonne Université, UVSQ, CNRS, Paris, France; 4 CNRS, CNES, UAR 2877 - ICARE Data and Services Center, Univ. Lille, 59000 Lille, France; 5 CNRS, UMR 8518 – LOA - Laboratoire d’Optique Atmosphérique, Univ. Lille, 59000 Lille, France; 6 European Centre for Medium-Range Weather Forecasts, Reading, RG2 9AX UK; 7 Spectroscopy, Quantum Chemistry and Atmospheric Remote Sensing (SQUARES), Université Libre de Bruxelles (ULB), Brussels, Belgium

**Keywords:** Climate-change impacts, Atmospheric dynamics, Atmospheric chemistry, Environmental monitoring

## Abstract

Biomass burning is the main source of air pollution in several regions worldwide nowadays. This predominance is expected to increase in the upcoming years as a result of the rising number of devastating wildfires due to climate change. Harmful pollutants contained in the smoke emitted by fires can alter downwind air quality both locally and remotely as a consequence of the recurrent transport of biomass burning plumes across thousands of kilometers. Here, we demonstrate how observations of carbon monoxide and aerosol optical depth retrieved from polar orbiting and geostationary meteorological satellites can be used to study the long-range transport and evolution of smoke plumes. This is illustrated through the megafire events that occurred during summer 2020 in the Western United States and the transport of the emitted smoke across the Atlantic Ocean to Europe. Analyses from the Copernicus Atmosphere Monitoring Service, which combine satellite observations with an atmospheric model, are used for comparison across the region of study and along simulated air parcel trajectories. Lidar observation from spaceborne and ground-based instruments are used to verify consistency of passive observations. Results show the potential of joint satellite-model analysis to understand the emission, transport, and processing of smoke across the world.

## Introduction

Wildfires are a common occurrence around the world and are an essential part of some ecosystems. However, several regions worldwide are experiencing increasingly devastating wildfire seasons due to the changing climate^1^. The smoke emitted by these fires is particularly harmful for humans, as it contains a cocktail of gases and particulate matter including carbon monoxide (CO) and fine particulate matter (PM2.5)^2^, which can penetrate into the lungs and enter the bloodstream^3,4^. By analyzing 65 million deaths in 43 countries, Chen et al.^5^ found an increasing risk of death from all causes soon after exposure to wildfire smoke, which was found responsible for tens to hundreds of thousands of premature deaths around the world each year.

The Western United States (US) are strongly affected by the increase in wildfire occurrence and resulting burned area^6^. This is linked to climate factors^7^, together with changes in landscape conditions and fire regimes^8^. Strong winds and lightning storms combined with increasingly frequent droughts also contribute to fire growth. It has been shown that the US summer wildfire season is already 20–40 days longer on average than it was 40 years ago^9^. As fire activity intensifies in the American West the impact on air quality was reported to grow due to the higher altitude reached by smoke and the greater injection of aerosols aloft^10^. In parallel, 6300 deaths per year have been associated with chronic exposure to smoke between 2006 and 2018 only in the US^11^. Finally, recent studies have shown that rising emissions from wildfires are counteracting the reductions in human-produced aerosol pollution over North America^12^.

Operational forecasting systems use estimated emissions from wildfires and can be used to predict their impact on local and remote air quality. The accuracy of the atmospheric models used in these forecasting systems in predicting smoke transportation at regional and global scales is impacted by the limited knowledge on a number of factors of which fire emission estimates and numerical diffusion are particularly prominent^13^. Although operational forecasts already rely on satellite observations for their initialization, spaceborne remote sensing can further contribute to reducing some of these uncertainties thanks to the global coverage, near-real-time availability, and improving sensing capabilities of meteorological space missions.

**Figure 1:**
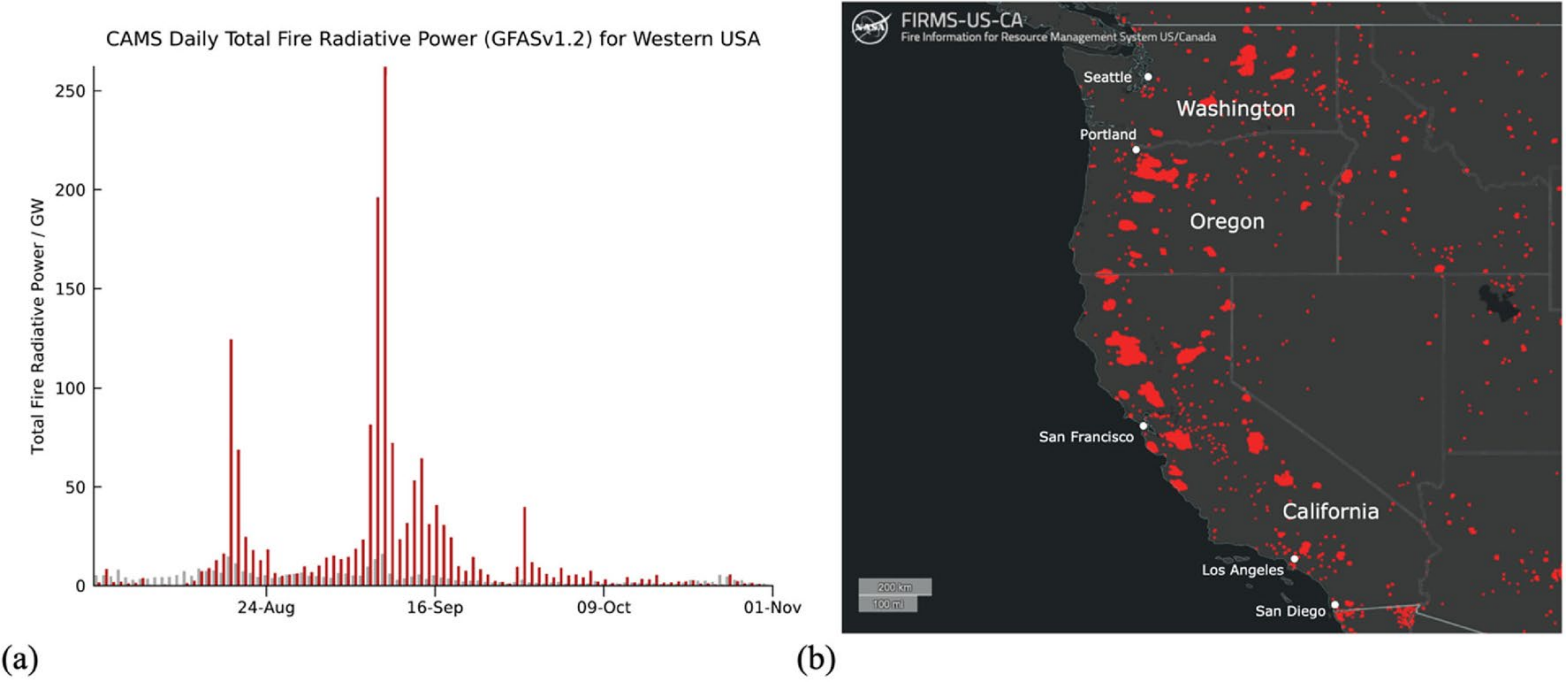
(**a**) Daily estimates of total fire radiative power from CAMS GFAS for Western US (including the states of California, Oregon, and Washington). Red bars correspond to the values corresponding to the 2020 wildfire season while gray bars give the average fire radiative power over the 2003–2019 period. (**b**) Fires detected by VIIRS/NOAA20 from August 19th to September 18th, 2020, in Western US. Image credits: Fire Information for Resource Management System US/Canada.

The aim of this article is to demonstrate the potential of satellite remote sensing to improve our understanding of emission, transport, and evolution of biomass burning aerosols. Here we focus on the Western US wildfire season of 2020, which was the largest recorded in California’s modern history with the burning of 4% of the state (https://www.fire.ca.gov/incidents/2020/). The scale of the fire activity can be reflected in daily totals of fire radiative power, a measure of radiative heat output that is observed by several satellite missions, that were several times larger than the 2003–2019 average throughout much of the summer of 2020 according to estimations from the Copernicus Atmosphere Monitoring Service (CAMS) Global Fire Assimilation System (GFAS)^14^ as shown in Fig. 1a. Fire activity peaked in late August and the first half of September, with multiple hotspots in the states of California, Oregon, and Washington according to the Visible Infrared Imaging Radiometer Suite (VIIRS) on NOAA-20 as seen in Fig. 1b. The analysis of the smoke emitted by these fires and its easterly transport across the Atlantic Ocean is performed using state-of-the-art measurements of (1) CO total column provided by the space-based Infrared Atmospheric Sounding Interferometer (IASI) instruments on polar orbiting platforms and (2) aerosol optical depth (AOD) provided by a combination of optical imagers on five different geostationary platforms. Satellite data are compared with analyses of CO total column and AOD from the CAMS operational system to check how well the model represents trace gases and aerosols during the emission and long-range transport of biomass burning plumes. In addition, lidar observations from the spaceborne Cloud-Aerosol Lidar with Orthogonal Polarization (CALIOP) and ground-based instruments in Europe are used to verify the consistency of remotely sensed passive observations and model outputs at different stages during the transport of smoke, especially with respect to their altitude. Finally, simulated forward and backward air parcel trajectories from the Hybrid Single-Particle Lagrangian Integrated Trajectory (HYSPLIT) model are used to monitor the characteristics and the temporal evolution of the smoke plumes from the emission locations to Europe.

## Results and discussion

### Monitoring smoke plumes with satellite and model data

Among the numerous trace gases emitted by fires, CO is directly associated with incomplete combustion^15^ and is a good proxy for smoke due to its long lifetime^16^ and its insolubility^17^. Biomass burning plumes are also associated with an increase in AOD with respect to clear conditions. The relation between these two variables in some conditions was shown in previous works on biomass burning emissions^18,19^ or the use of CO as a proxy for aerosols^15,20^, including studies about aerosol-cloud processes^21,22^.

**Figure 2:**
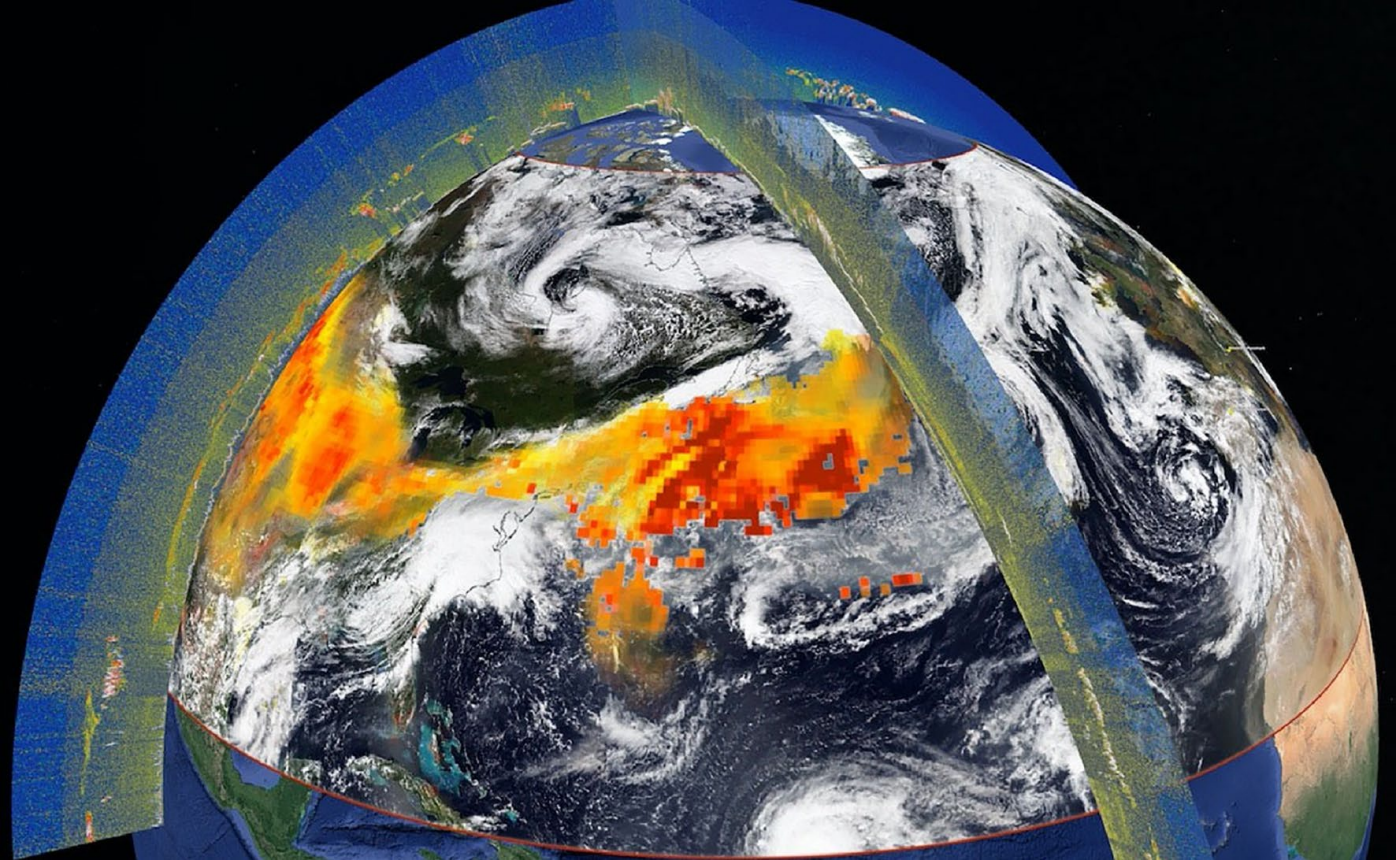
Illustration of the smoke plume observed on the 17th of September, 2020. The total AOD retrieved from GEO-ring is depicted in shades of yellow (low AOD) to red (high AOD). The VIIRS true color image in the background enables to see the large-scale atmospheric circulation in which the plume is being transported. Two vertical curtains illustrate the CALIOP total attenuated backscatter observations used to determine aerosol plume altitude. More detailed CALIOP illustrations, including vertical feature masks, for the most intense parts of the plumes are provided as Supplementary Fig. S1 and Fig. S2 online.

In this study, we use daily maps of CO total columns provided by the IASI instruments on the Metop polar orbiting satellites (Metop-A, B, and C) and total AOD at 640 nm derived from a constellation of geostationary satellites referred to as GEO-ring (see “Methods” section for a full description of these products). These global data sets are analyzed to track the advection of the smoke emitted by the Western US wildfires in September 2020, which crossed the Atlantic on several occasions. Smoke pollutants are indeed often transported downwind over large distances due to their injection into the upper troposphere^10^ due to pyro-convection generated by large fires. At these altitudes, the photochemical lifetime of smoke is longer, winds are stronger, and particles are further away from the surface and precipitation, which minimizes dry and wet deposition respectively. The region of study was therefore defined to encompass the trajectory of smoke from Western US to Europe. Satellite observations were interpolated to account for their different spatial and temporal characteristics and to provide one observation per day (see “Methods” section for details on the data processing). It is important to note that AOD and CO total columns cannot be retrieved from the satellite observations if clouds are present. The analysis of smoke transport is also conducted using analyses of these two quantities provided by the CAMS system, which operationally assimilates satellite observations of atmospheric composition including IASI CO (but excluding GEO-ring AOD). Figure 2 illustrates the thick and extended aerosol plume observed on September 17th, 2020, as it was transported from Western North America to Eastern North Atlantic, using the GEO-ring AOD product along with two segments of CALIOP orbits. The existence of high AOD values according to GEO-ring coincide with the presence of aerosol layers detected by CALIOP (at an altitude of 4 km approximately in the instance of the orbit across the Atlantic for example) as shown in Fig. 2 and in Supplementary Fig. S1 and Fig. S2 online. The true color composite in the background depicts the synoptic situation on that day as observed by VIIRS/NOAA-20.

**Figure 3:**
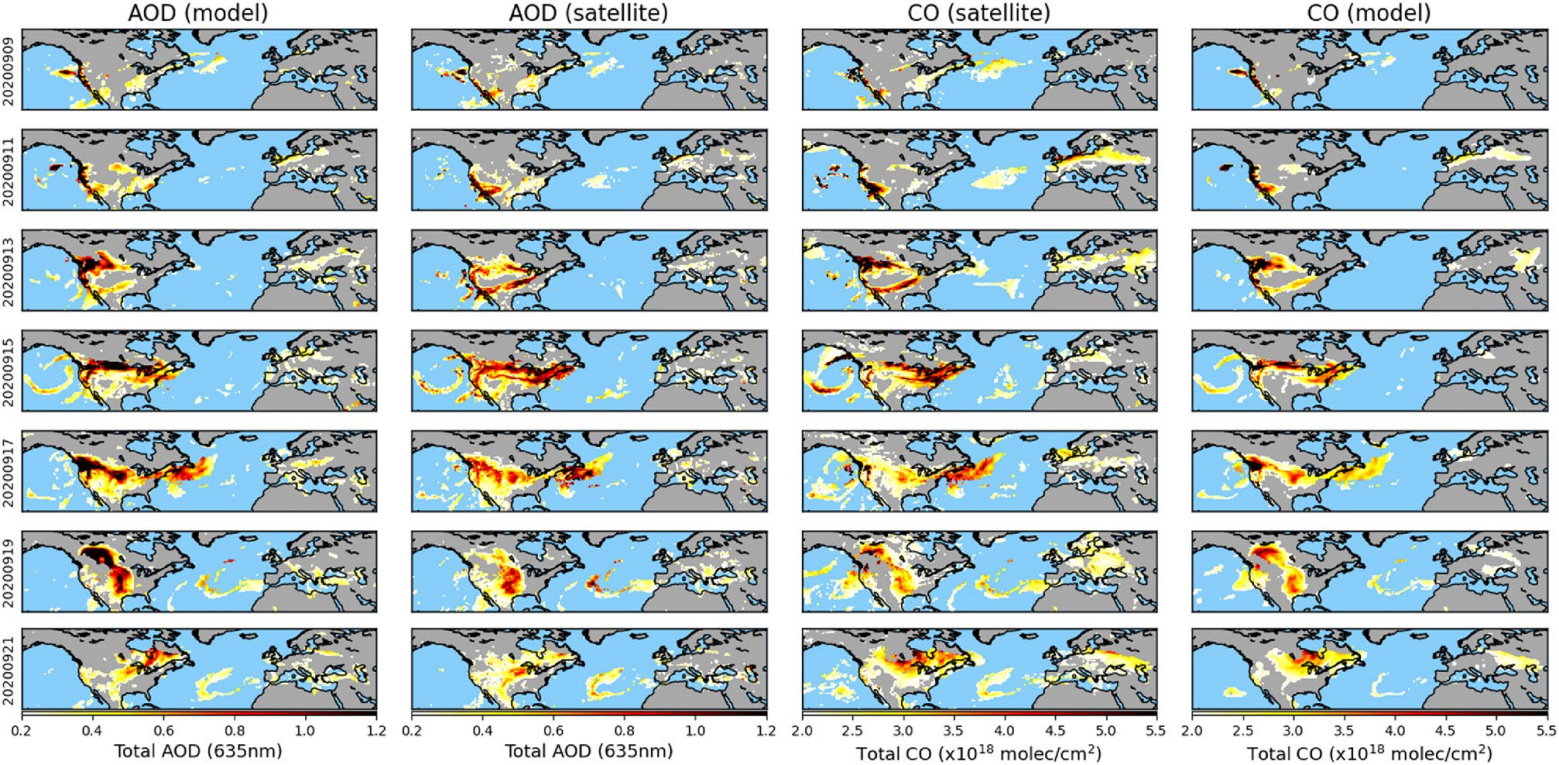
Daily evolution of CAMS AOD, GEO-ring AOD, IASI CO, and CAMS CO, for selected days in September 2020. Note that AOD and CO are shown for values larger than 0.2 and 2 × 10^18^ molec/cm^2^, respectively, to highlight thick aerosol plumes only. Regions corresponding to heavy cloud cover and coarse aerosol particles were masked in the data preprocessing.

Figure 3 illustrates the temporal evolution of smoke plumes across the region of interest for a selection of representative days in September when the highest smoke activity was observed. An extended timeline spanning the most part of the month with a step of 1 day can be found as Supplementary Fig. S3 online. Daily maps of AOD and CO total columns observed by satellite and from the CAMS analyses show the intense emission of smoke and biomass burning aerosols along the western coast of the US. The emission peak is observed on September 11th and 13th, with values of CO and AOD surpassing 30 × 10^18^ molecules/cm^2^ and 3, respectively. Long-range transport is observed until the 21st, with massive plumes traveling from Western US to the Atlantic Ocean. Smoke from a previous peak in emissions is observed to reach the northwest of Europe on September 11th (see Fig. 3 and Supplementary Fig. S3 online) after it had been transported over more than 9000 km. On this date, transported CO and AOD attained values of 4 × 10^18^ molecules/cm^2^ and 0.4 in average along the coast of Belgium and the Netherlands. Overall, all four datasets show a good agreement throughout the selected dates despite coming from different sources (model versus satellite) and representing different physical quantities (CO total column versus AOD). Some minor differences can be however observed in Fig. 3 on the location, shape, and magnitude of some smoke plumes. These dissimilarities are discussed in the following section. It is worth mentioning that while the assimilation of IASI retrievals into the CAMS model explains in part the good agreement between the two CO data sets, we observe some differences that may come from the assimilation of CO observations from other satellites (see “Methods” section) and the weight of the initial state of the model analysis in the production of the eventual analyses.

### Data assessment during the intercontinental transport of smoke

**Figure 4:**
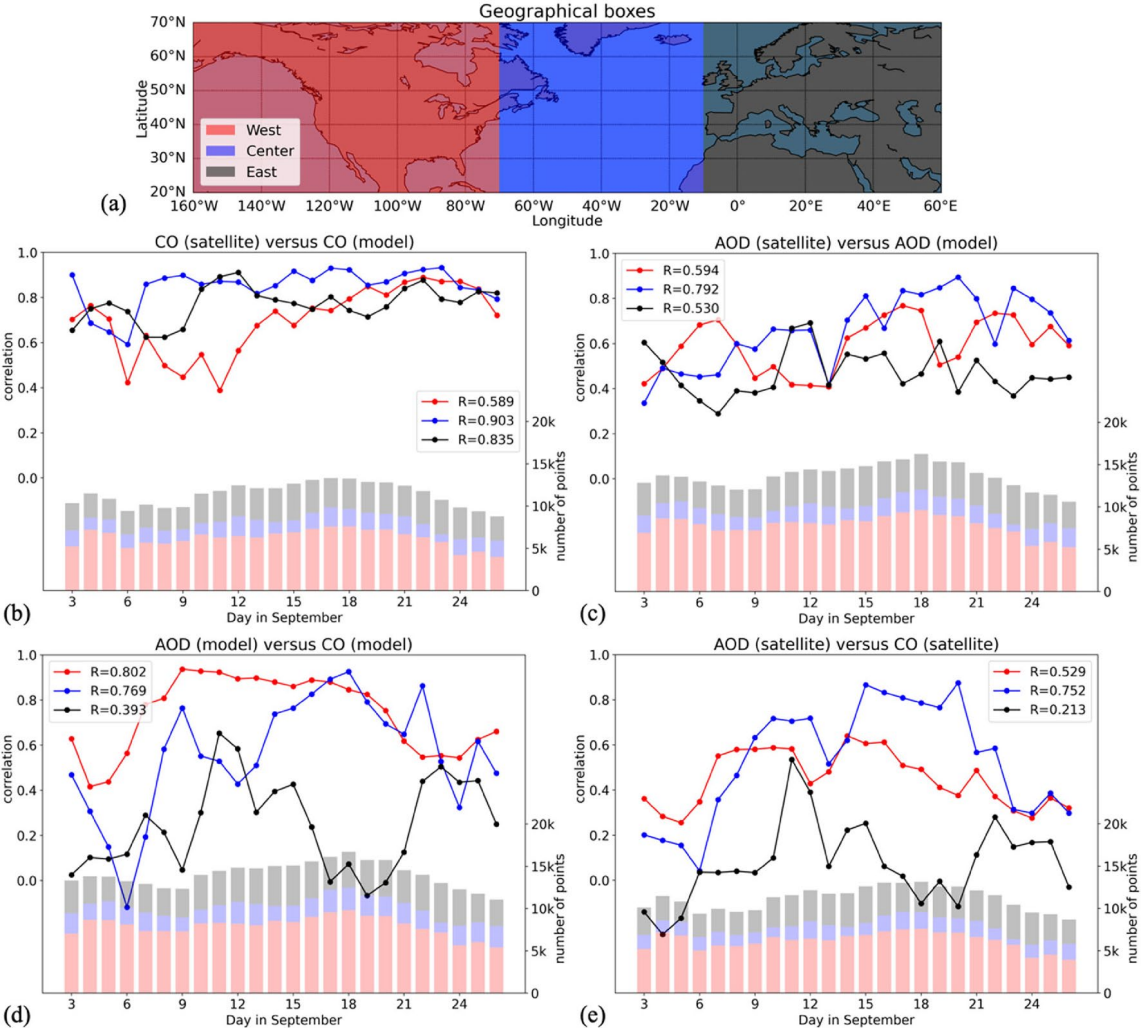
(**a**) Geographical domains used in the study. Daily correlation during the period of study for each domain between (**b**) IASI CO and CAMS CO, (**c**) GEO-ring AOD and CAMS AOD, (**d**) CAMS AOD and CAMS CO, (**e**) GEO-ring AOD and IASI CO. Average Pearson correlation coefficient (R) is given in the legend for each domain. The height of bars associated to the right y-axis are representative of the total number of pixels that were used for comparison, with colors being used to divide this number into the three domains. Colors in (**a**) correspond to the bars and plots in the other charts. The lower number of points for the Central domain comes from the smaller spatial extent compared to the others domains and the higher occurrence of clouds over ocean than over land.

We further assess the agreement among the four datasets by inspecting their mutual correlation over three geographical domains (hereafter referred as West, Central, and East) that were defined according to longitude as shown in Fig. 4a. The period of study is extended here from September 3rd to September 26th, 2020.

Satellite retrievals are first compared to CAMS analyses, in terms of CO total columns in Fig. 4b and AOD in Fig. 4c. Regarding CO, IASI and CAMS agree well with each other with an average correlation scores of 0.90 and 0.83 for the Central and East domains, respectively. A lower correlation (average of 0.59) is however observed in the West domain, especially during the first half of the period of study, which could be associated with the generally higher biases in the two data sets in the occurrence of intense smoke emission. While errors in the CAMS analyses may be related to uncertainties in the estimates, injection height, and diurnal cycle of fire emissions considered in the model, satellite observations may be biased due to the erroneous cloud masking of thick smoke plumes. Regarding aerosol load, the GEO-ring AOD product shows a fairly good agreement with CAMS analyses (average correlation between 0.53 and 0.79) despite being two unconnected datasets (i.e., GEO-ring AOD is not assimilated in CAMS) and the shorter lifetime of AOD compared to CO. The maximum correlation value of 0.88 is observed for the Central domain around September 19th, when vast smoke plumes were traveling across the Atlantic (see Fig. 3), which validates the way the transport of plumes is implemented in the model.

CO and AOD are compared in Fig. 4d for CAMS and in Fig. 4e for the satellite products. The three domains are inspected sequentially. First, the CAMS analyses in the West domain show a high correlation (average of 0.80) between CO and AOD, peaking during the dates of maximum aerosol loading. However, the satellite data show a generally lower correlation (average of 0.53) for the West domain, which may come from specific limitations of the retrieval processes (e.g., cloud filtering) or the different overpass times. Second, high correlation between CO and AOD can be observed in the Central domain, especially from September 14th to 21st for both CAMS (average of 0.77) and the satellite products (average of 0.75). This comes from the transport of huge plumes across the Atlantic Ocean as seen in Fig. 3. Finally, results for the East domain show a generally lower correlation (average of 0.39 and 0.21 for CAMS and satellite data, respectively), potentially coming from the contribution to AOD of other aerosol species in addition to smoke. However, several correlation peaks between AOD and CO are observed on September 11th, 15th, and 22nd, which we attribute to the arrival of biomass burning smoke plumes in Europe. These findings are reported both from satellite and model data, underlining again the consistency among data sets.

### Local detection of smoke plumes in Europe

**Figure 5:**
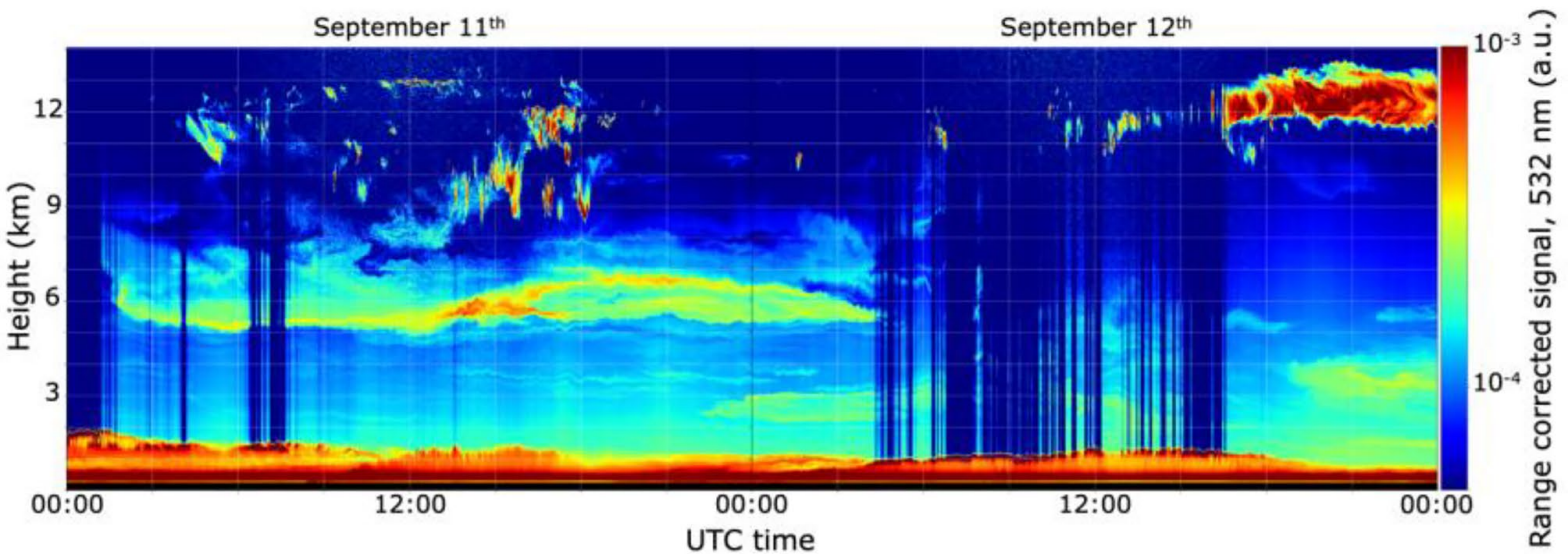
Vertical profile of range-corrected lidar signal at 532 nm (in arbitrary units; a.u.) on September 11th and 12th, 2020, from METIS lidar in Villeneuve d’Ascq, France.

The arrival of smoke plumes in Europe was observed in September by several ground-based lidar stations. For example, the ground lidar in Leipzig, Germany, detected high concentrations of smoke on September 11th in agreement with measurements from the Aeolus wind lidar mission^23^. This plume was also detected by lidar observations from the Aerosol Clouds and Trace Gases Research Infrastructure (ACTRIS) network. Figure 5 shows the presence of smoke also on September 11th from the ground-based METIS lidar over Villeneuve d’Ascq, 200 km north of Paris in France. This station measured an aerosol layer extending between 5 and 7 km on the 11th, which remained visible on the 12th. From the same lidar station, several smoke plumes, similar to the one visible from Fig. 5, were detected on September 14th, 17th, and 18th (not shown here).

**Figure 6:**
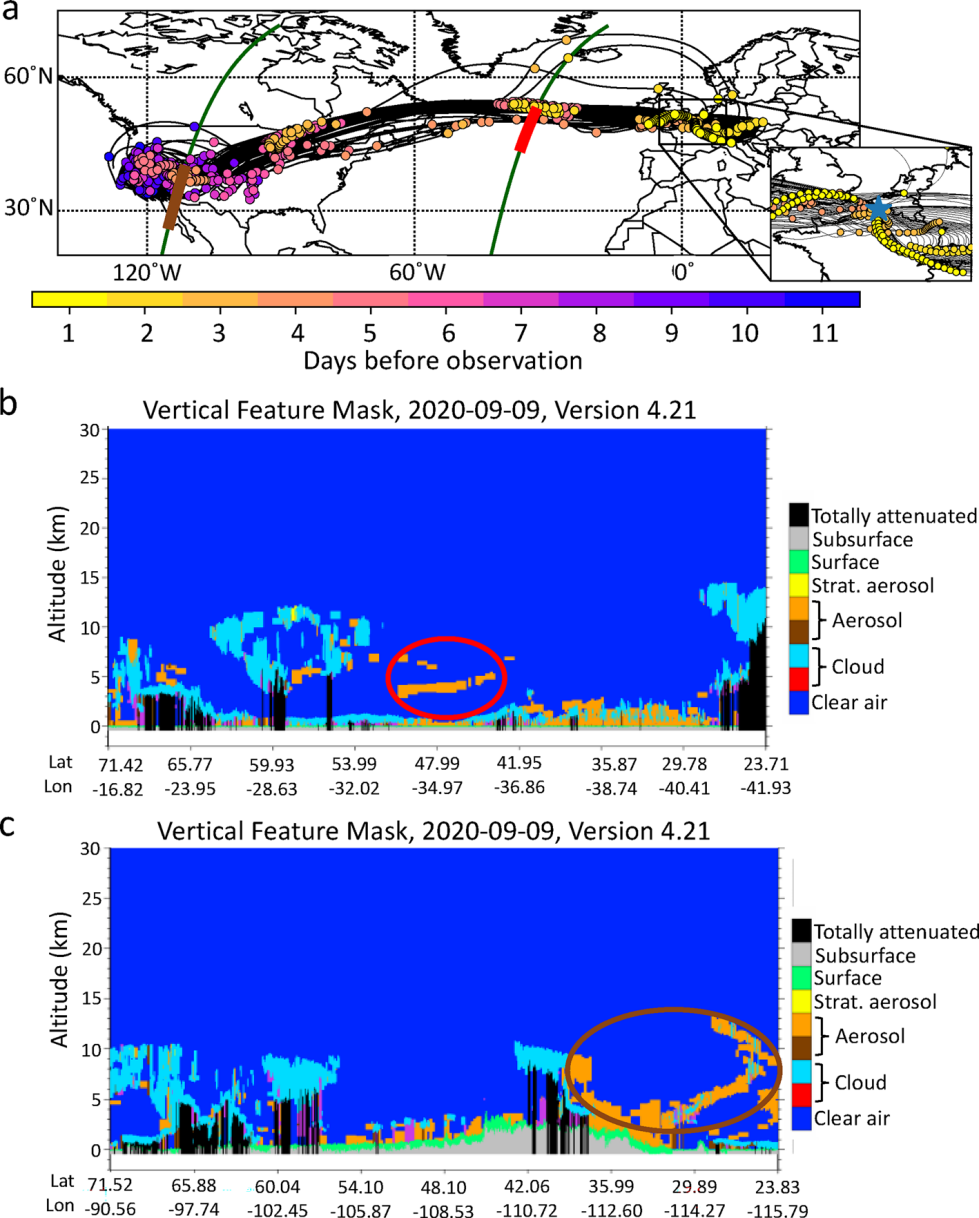
(**a**) Back trajectories initialized from smoke plumes observed over Villeneuve d’Ascq (located with a blue star in the zoomed area box). The colored dots represent the number of days before the observations at 12 UTC. The green lines represent two tracks from the space-based lidar CALIOP from September 9th, 2020, associated with biomass burning plumes for which four back trajectories have been collocated. The red and brown parts on the track represent the region where the plumes were detected. Subplots (**b**) and (**c**) represent the vertical feature mask estimated from CALIOP of the two profiles from subplot (**a**) and the biomass burning plumes are circled with the corresponding color. Image credits: NASA LaRC https://www-calipso.larc.nasa.gov/.

We analyze air parcel back trajectories generated from the HYSPLIT model for the four plumes observed on September 11th, 14th, 17th, and 18th. To define back trajectories starting points, a time-height grid was set over lidar profiles obtained at Villeneuve d’Ascq ground site. Detected plumes were gridded with a temporal and altitude resolution of 1 h and 500 m, respectively. Each grid cell was then used to initialize calculation of a given back trajectory. In total, 303 back trajectories were established but only those tracing back to Western US are considered here (corresponding to 27% of the dataset). Figure 6a shows the back trajectories with an indication along the paths of the elapsed time to their detection by the ground lidar station (i.e., when the back-trajectories are initialized). The transport time of the air parcels from their emission in Western US to their detection over Europe ranges from 3 to 12 days. The fastest air parcels take 1 day to be transported from the source region to the Atlantic and the slowest air parcels take up to 8 days for the same journey. Then, air parcels take between 2 and 6 days to be transported from the Atlantic Ocean to Villeneuve d’Ascq where they are detected. From the zoomed area box in Fig. 6a, we observe that 40% of the selected back trajectories arrive in Villeneuve d’Ascq from Eastern Europe, 4% from the north, and the majority (56%) from the west.

Figure 6b, c show the vertical feature masks estimated from two CALIOP tracks on September 9th for which smoke aerosol plumes have been detected (in orange color). Four back trajectories from Fig. 6a are found to be collocated with the two CALIOP vertical profiles, one collocated with the plume detected over the Atlantic (Fig. 6b) and three collocated with the plume detected over southwestern US (Fig. 6c). The spatiotemporal location of the back trajectories are geographically close to the CALIOP observations of the plumes. For example, differences were found to be lesser than 2 h in time, 2 km in height, 3° in latitude, and 5° in longitude for the plume detected over the Atlantic (Fig. 6b). It is worth noting however that this evaluation may be affected by numerical and physical uncertainties of the HYSPLIT model as discussed in previous studies^24^. The synergy of the lidar profiles with the back trajectories confirms that the plumes detected over the Atlantic Ocean and the Western US coast were transported to Europe.

### Evolution of AOD and CO along smoke trajectories

**Figure 7:**
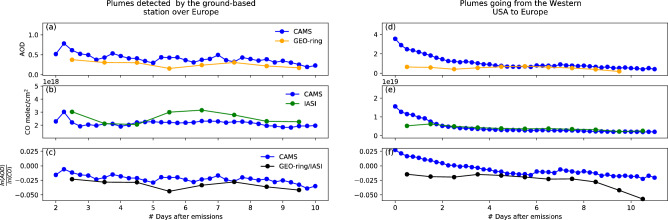
Temporal evolution of (**a**) AOD, (**b**) CO, and (**c**) the ratio of the logarithms of both quantities of smoke plumes along back trajectories from Western US to their detection in Europe considering a transport lasting 8 days. The dots represent the mean as a function of the number of days since air parcels leave Western US. The shaded areas are delimited by the standard deviation. Subplots (**d**), (**e**), and (**f**) are similar to subplots (**a**), (**b**), and (**c**) but correspond to air parcels along forward trajectories with an AOD greater than 2.3 over the Western US and transported to Europe.

Figure 7a, b show the mean temporal evolution of AOD and CO from the satellite observations and CAMS analyses along the back trajectories. To limit the impact of the potential uncertainties from HYSPLIT, we considered the means of several trajectories to infer the results. To avoid mixing signals from very fast or slow transport, we only take into account back trajectories that lasted 8 days from the time the air parcels leave the Western US to their detection over Europe. The absolute values are different for faster or slower transports, but the general trends are similar and the number of data points is the highest considering 8 days allowing robust statistics (not shown). Here, we use the daily AOD and CO values from GEO-ring and IASI while CAMS atmospheric analyses are used at their original temporal resolution of 6 h (see “Methods” section for details on the data). From Fig. 7a, we observe that the AOD from CAMS differs from the satellite observations by about 0.14 but both show a consistent decrease in AOD with time. Indeed, the mean AOD ranges from 0.5 and 0.4 for the analyses and the observations, respectively, when the air parcels leave Western US to 0.2 for both datasets when the air parcels are observed over Europe. From Fig. 7b, we observe that CO from CAMS differs from the satellite observations by about 4.42 × 10^17^ molecules/cm^2^. Similar to AOD, CO decreases over time along transport, from 3.02 × 10^18^ to 2.26 × 10^18^ molecules/cm^2^ for IASI and from 2.30 × 10^18^ to 1.96 × 10^18^ molecules/cm^2^ for CAMS.

Figure 7c shows the ratio of the logarithms of AOD and CO along the back trajectories from Western US to Villeneuve d’Ascq in Northern France. The logarithm is used here to make these two quantities more comparable. While the ratio is overestimated by CAMS analyses by 0.010 compared to the satellite observations, both datasets show a ratio that slightly decreases over time. For CAMS (satellite observations), the mean ratio decreases from − 0.016 (− 0.023) to − 0.035 (− 0.042).

We finally analyze 200 forward trajectories that were computed for air parcels in September 2020 associated with a CAMS AOD greater than 2.3 over the Western US, corresponding to the upper quartile of AOD for that region. This threshold is applied to ensure that the air parcels are initially associated with a significant biomass burning plume. Figure 7d–f show the temporal evolution of AOD, CO, and the ratio of the logarithms of AOD and CO for air parcels along forward trajectories that originated from Western US and were transported to Europe. The low values of the mean AOD when air parcels arrive over Europe suggest that aerosols have been removed or dispersed during the transport for most of the trajectories, unlike Fig. 7a–c. Figure 7d shows that AOD decreases by an absolute value of 3.1 over time for CAMS and by 0.5 for the satellite data. Figure 7e shows that CO decreases by 13.6 × 10^18^ molecules/cm^2^ for CAMS and by 3.0 × 10^18^ molecules/cm^2^ for the satellites. Finally, Fig. 7f shows that the ratio of the logarithms of AOD and CO decreases by 0.047 for CAMS and by 0.043 for the satellites. The trends from Fig. 7d–f are the same as for Fig. 7a–c but the differences in the AOD, CO, and the ratio of both over time are larger. The similarities in the temporal variation of CO and AOD and the ratio of the two quantities show that the analysis from CAMS can accurately reproduce processes of dispersion of gases and aerosols with respect to the satellite data used in this work. However, we cannot determine, from this analysis only, whether biomass burning aerosols are dispersed by dilution^25^ or scavenged by precipitation en route^26^.

### Precipitation occurrence along long-range transport of smoke

Biomass burning aerosols transported from Western US to Europe may have their concentration decreased due to precipitation, dry deposition, or by diffusion. On the other hand, CO is not affected by rain and its concentration only decreases due to dispersion, acting as a passive tracer of originally polluted air parcels^15^. A sudden decrease in the ratio of AOD and CO in the case of smoke plumes could therefore be explained by a rapid removal of aerosol particles which in turn is most likely to be caused by precipitating clouds at or above the smoke plume level. Contrarily, if the ratio remains constant or increases, it could mean that the trajectory from the source region to the observation station is not associated with aerosol removal which would lead to a longer aging time of the plume and potentially longer interaction of these aerosols with clouds on their way.

Figure 7a–c are based on back trajectories initialized from aerosol plumes detected by the lidar in Villeneuve d’Ascq. The presence of smoke particles over Europe demonstrates the absence of efficient aerosol removal (e.g., precipitation) during its transport from Western US. A decrease is still observed in the ratio of AOD and CO in Fig. 7c which can be associated to the dispersion of AOD and/or CO, or the mixing of air parcels at different altitudes and therefore subject to different vector winds. These possibilities are supposed to affect forward trajectories in the same way. Nonetheless, the decrease in the ratio over the full temporal window is 2.5 times larger in Fig. 7f (0.047 for CAMS) than in Fig. 7c (0.019 for CAMS). The air parcels from Fig. 7f are associated with biomass burning plumes over the Western US but the AOD and CO are low over Europe. Most of these trajectories are therefore associated with a more active aerosol removal occurring during transport, which does not allow biomass burning plumes from Western US to reach Europe. While decorrelation between CO and aerosols may also occur during transport for other reasons, the IMERG^27^ (Integrated Multi-satellitE Retrievals for Global precipitation measurement mission) daily accumulated precipitation product analyzed for September 2020 indicates that significant precipitation events occurred frequently on the preferential transport route from Western US to Europe, pointing to a high probability of interactions between precipitation and smoke plumes studied herein.

## Conclusions

The increasing number and intensity of wildfires worldwide induced by global warming have devastating ecosystem, social, and economic impacts. Biomass burning is nowadays identified as the predominant source of air pollution in many regions of the world including large parts of Canada, Siberia, Africa, South America, and Australia^28^. Emitted smoke plumes can be transported across long distances and affect the local air quality in downwind regions thousands of kilometers away, which makes it essential to better understand their transport. In the summer 2020, wildfires burning in Western US carried harmful pollutants across the country and reached Europe as demonstrated for example by local lidar station measurements.

This study illustrates the usefulness of satellite and model data to monitor the rapid intercontinental transport of smoke plumes through the joint use of total AOD and CO total columns. Information provided by the GEO-ring (a constellation of geostationary optical imagers) and IASI on the transport of smoke plumes in September 2020 proved to be generally similar with respect to CAMS analyses, merging satellite observations with global models, albeit some differences were observed. The disagreement, which was found to be higher during the intense emission of smoke, might be caused by biases in the satellite retrieval algorithms, the timing of the satellite overpasses in relation to the time of the assimilation window in CAMS, or limitations in the fire emission estimations used in the model. The last point could be better understood in a follow-up study using CAMS outputs with no satellite data assimilation. Alternatively, assimilation of the GEO-ring satellite observations used in this work is certainly one potential way to overcome these limitations by providing improved model analyses. Furthermore, upcoming observations of fire smoke at high temporal resolution from the new generation of geostationary satellites^29^ are expected to lead to improvements in forecasting.

Back trajectories of smoke plumes detected over Europe enable the analysis of the temporal evolution of AOD and CO during the transport. The temporal variation of the AOD to CO ratio during the transport of biomass burning from Western US is smaller for plumes that are detected over Europe than for plumes that are potentially not. We hypothesize that the difference is most likely associated to a more effective aerosol removal process along transport and suggest that this removal is primarily due to cloud formation or precipitation in accordance with previous works^30–32^. Therefore, analysis of atmospheric components retrieved by satellite measurements or models could potentially be used to infer information on cloud processes acting on aerosols. Inconsistent AOD to CO ratio estimated from satellites and models could point to weaknesses in model representation of precipitation and other aerosol removal processes. The present results indicate that a systematic analysis of AOD and CO observed by satellites and models could help to study the emission and transport of smoke plumes in agreement with previous studies^18,20^, but also to infer information on precipitation and evaluate model performance at representing wet scavenging processes.

## Methods

### CO satellite data

Carbon monoxide is a key species for tracking pollution plumes and atmospheric dynamics. The background CO atmospheric loading varies as a function of season and latitude and is significantly perturbed by human activities related to combustion processes, including large fires. In this study, CO total columns are derived from the nadir radiance observations provided by the IASI suite of satellites^33^. The IASI mission flies onboard the sun-synchronous polar-orbiting Metop platforms, crossing the equator at around 9:30 (day) and 21:30 (night) local time. It uses a nadir viewing geometry to record atmospheric spectra in the thermal infrared spectral range, from which trace gases concentration can be retrieved^34^. To retrieve CO, we take advantage of absorption in the fundamental 1–0 CO rotation-vibration band centered around 4.7 µm, using the Forli 2019 retrieval algorithm^35^. Previous studies have shown that the maximum sensitivity is for the mid-troposphere, and that total column (i.e., the vertically integrated concentration) is a robust product to track smoke transport^36^. Previous validation studies using ground-based, aircraft, and satellite data have shown that CO total columns from IASI are retrieved with an error generally below 10–15% at mid and tropical latitudes but can have larger errors in polar regions. As clouds in the field of view can obstruct or reduce the visibility and prevent observation of the lower layers of the atmosphere, only data with cloud contamination less than 25% were selected.

### AOD satellite data (and cloud cover)

AOD is a measure of the extinction caused by aerosols along the vertical path from Earth’s surface to outer space and is widely used as a proxy of particle content. In this study, measurements of total AOD (coming from all types of particles suspended in the atmosphere) are provided from a constellation of five geostationary meteorological satellites named GEO-ring. This includes the American Geostationary Operational Environmental Satellites (GOES)-17 and GOES-16^37^, the European Meteosat-11 and Meteosat-8 satellites^38^, and the Japanese Himawari-8 satellite^39^. The combination of the optical imagers aboard these platforms allows for global coverage thanks to their evenly spaced position around the globe (at 137.2°W, 75.2°W, 0°, 41.5°E, and 140.7°E, respectively). Several times per hour, each satellite acquires an image of the Earth’s disk observed from its position along the geostationary orbit, at several visible and infrared wavelengths, and at a spatial resolution between 1 and 3 km. The high number of images per day provided by geostationary satellites allows for a robust detection of aerosols^40^. Global maps of daily-averaged total AOD at 640 nm at 0.1° of resolution were retrieved from the GEO-ring following Ceamanos et al.^41^. This was achieved by using the Aerosol and surface albEdo Retrieval Using a directional Splitting method-application to GEOstationary data (AERUS-GEO) algorithm^42^. GEO-ring retrievals were found to be accurate with respect to collocated ground aerosol measurements from the Aerosol Robotic Network (average correlation of 0.76 and mean bias error of 0.01). Moreover, the spatial and temporal coverage of this product was proved to be greater than traditional aerosol products derived from polar orbiting satellites like MODIS^43^.

Information on cloud cover is obtained using the software from the Satellite Application Facility on Support to Nowcasting and Very Short Range Forecasting^44^. The application of this cloud detection method to the GEO-ring data and the posterior combination of the resulting binary masks into a daily composite provided global maps of cloud fraction between 0 and 1, with 1 corresponding to the presence of clouds throughout the day.

### CAMS analyses

CAMS is operated by the European Center for Medium-Range Weather Forecasts and provides near-real time analyses and forecasts of atmospheric composition using the integrated forecasting system^45^ (https://www.ecmwf.int/en/publications/ifs-documentation) including the sources, sinks and tendencies for atmospheric chemistry. In the present study, we consider the vertically integrated CO total columns and total AOD at 550 and 670 nm^46,47^ from the CAMS operational analysis with a spatial resolution of 0.4° in latitude and longitude and a temporal resolution of 6 h. The CAMS system assimilates satellite observations of atmospheric composition including AOD from MODIS^46^ and the Polar Multi-Sensor Aerosol product based on GOME-2/AVHRR, and CO from IASI^33^ and from the Measurement Of Pollution In The Troposphere (MOPITT)^48^. CAMS data are open access and freely available from the Atmosphere Data Store at https://ads.atmosphere.copernicus.eu/cdsapp#!/dataset/cams-global-atmospheric-composition-forecasts?tab=overview. CO from CAMS was found to be in good agreement when compared with continental measurements^49^ and shipborne measurement^50^. The AOD from CAMS has also been validated on several events. For example, Akritidis et al.^51^ found a correlation coefficient of 0.77 when comparing CAMS AOD with MODIS, CALIOP, and ground-based observations. CAMS analyses of AOD were recalculated at 640 nm to match the wavelength of the GEO-ring satellite retrievals using the Ångström exponent between 550 and 670 nm calculated from CAMS. In this study, analyses (at T + 0) were preferred over forecasts (at T + *x*) to prove the usefulness of assimilating additional satellite observations (at T + 0 as well) in the CAMS operational system.

### Methodology for data analysis

All above-mentioned data were made available from the 3rd to the 26th of September 2020. Data sets of IASI CO, GEO-ring AOD, and CAMS analyses of AOD and CO were collocated in time and space. This was done by averaging the IASI CO data over a 0.5° × 0.5° grid on a daily basis. CAMS AOD and CO were linearly interpolated a 0.5° × 0.5° grid. Satellite AOD and cloud fraction maps were down sampled by block averaging to match the resolution of 0.5°. To account for the fact that GEO-ring AOD is derived from daytime measurements only, a weighting factor of 2 and 1 was applied to IASI data corresponding to 9:30 and 21:30, respectively, when doing the daily average. Analogously, 6-hourly analyses from CAMS were interpolated to provide a single daily value centered at local noon. Satellite and CAMS data were cropped to the region of study that was defined from 160°W to 60°E and from 20°N to 70°N to encompass the Northeastern Pacific, North America, the Northern Atlantic, and Western Europe (see Fig. 4a). Cloudy regions were not considered in the data analysis by filtering pixels with a cloud fraction greater than 0.75. This was done to account for the fact that satellite estimates of CO and AOD are only available for clear sky conditions, contrary to model simulations which are available under all-sky conditions. Finally, only AOD values corresponding to fine aerosol particles (e.g., smoke) were considered in the data analysis. For this purpose, we discarded pixels corresponding to an Ångström exponent lower than 1. This filtering resulted in the removal of pixels containing coarse aerosol particles such as desert dust, which are not of interest in this study.

### Lidar observations

Lidar observations from spaceborne missions and ground-based systems are used in this study to confirm aerosol location (latitude, longitude, and altitude extend). This information is also used to initialize air parcel trajectories calculation. Two data sets are used:

- CALIOP/CALIPSO data. CALIOP is an active sensor flying aboard the CALIPSO (Cloud-Aerosol Lidar and Infrared Pathfinder Satellite Observations) platform as part of the A-Train satellite constellation since April 2006. It is a three-channel elastic backscatter lidar that measures high-resolution profiles of the attenuated backscatter from aerosols and clouds at visible (532 nm) and near-infrared (1064 nm) wavelengths along with polarized backscatter in the visible channel^52^. From these measurements can be derived aerosol backscatter and extinction profiles in both cloud-free and cloudy conditions^53^. Due to its active nature, CALIOP can be used during both day and night and provides an unprecedented and still unique view of the vertical distribution of aerosols and clouds in the troposphere. CALIOP native observations are acquired at high horizontal and vertical resolution (respectively 1/3 km and 30 m in low and middle troposphere). Based on these level 1 data, several clouds and aerosols products are derived at various resolution ranging from 1/3 km for basic quantities such as cloud mask profile up to 5 km for aerosol products such as AOD.
- METIS data. METIS is an automated micro-lidar operated by the ATmospheric Observations in LiLLe (ATOLL) platform of Laboratoire d’Optique Atmosphérique (LOA) in Villeneuve d’Ascq, France. The micro-lidar is a medium power CIMEL CE376 GPN instrument (https://www.cimel.fr/solutions/ce376) providing vertical extinction, backscatter and depolarization profiles up to 9 km during daytime and up to 18 km during nighttime.

### Air parcel trajectories

Back and forward trajectories of air parcels were performed by the Air Resources Laboratory’s HYSPLIT model^54^. HYSPLIT considers a Lagrangian approach to retrieve the advection and diffusion following the air parcel trajectories. The meteorological data fields from the NOAA-NCEP/NCAR pressure level reanalysis^55^ were used here to compute the dispersion module in the model with a 2.5° latitude–longitude projection. HYSPLIT trajectories are output with a 1-h interval.

### Supplementary Information

Supplementary Information.

## Data Availability

The IASI-CO used in this study are retrieved from the Aeris data infrastructure (https://iasi.aeris-data.fr/CO/). We used NCEP-NCAR Reanalysis 1 data provided by the NOAA PSL from their website at https://psl.noaa.gov. The CAMS data used are retrieved from the Copernicus Atmosphere Data Store (https://ads.atmosphere.copernicus.eu/). The GEO-ring AOD data used in this investigation can be accessed through the following link upon free registration on the ICARE Data and Service Center: https://www.icare.univ-lille.fr/data-access/data-archive-access/?dir=PUBLICATION_DATA/10.1029/2021JD034906/.
