# Supplementary material for: Remote sensing and model analysis of biomass burning smoke transported across the Atlantic during the 2020 Western US wildfire season
Source: Sci Rep. 2023 Sep 25;13:16014. doi: 10.1038/s41598-023-39312-1 (PMC10519943; doi:10.1038/s41598-023-39312-1)
Supplement: Supplementary file 1 — Supplementary Information. [file 41598_2023_39312_MOESM1_ESM.pdf]

# Supplementary Information of “Remote sensing and model analysis of biomass burning smoke transported across the Atlantic during the 2020 Western US wildfire season”

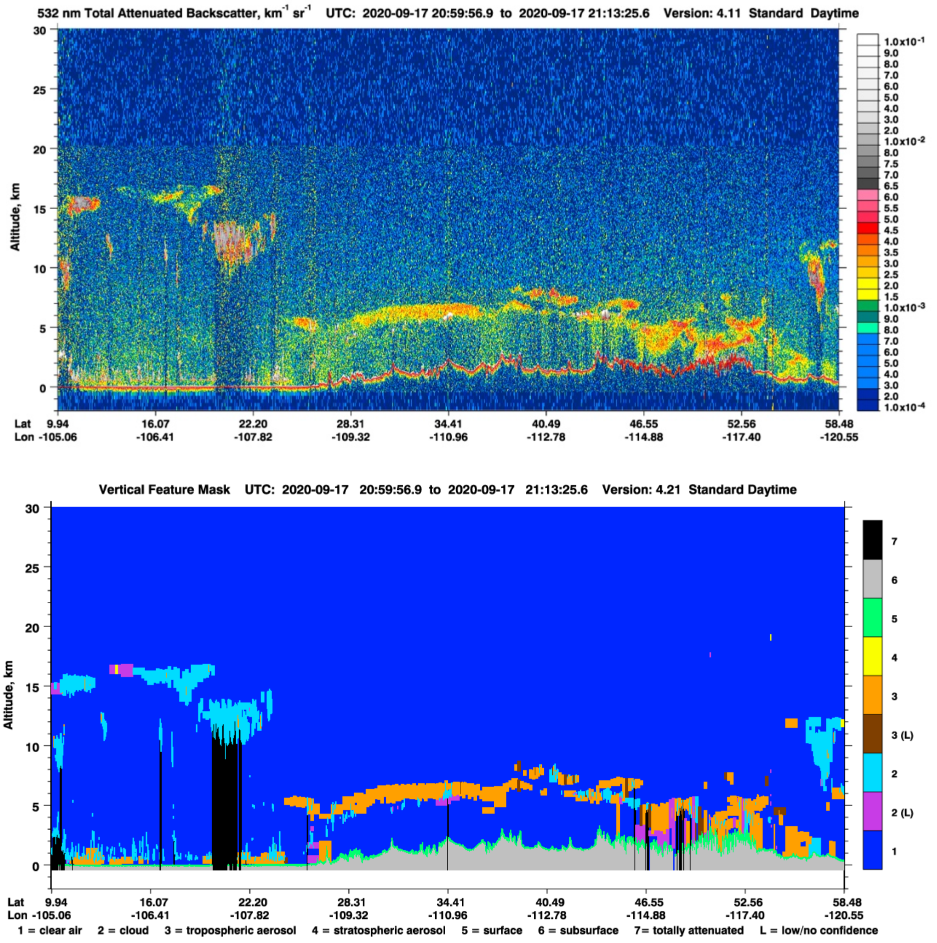


**Supplementary Figure S1.** Detailed views of the western CALIOP curtain used in Fig. 2. Only the midlatitude part of the curtain is depicted (where the aerosol plume was most intense). From top to bottom are presented the total attenuated backscatter signal and the vertical feature mask product with aerosol layers in orange color. Image credits: NASA LaRC [https://www-calipso.larc.nasa.gov/](https://www-calipso.larc.nasa.gov/).

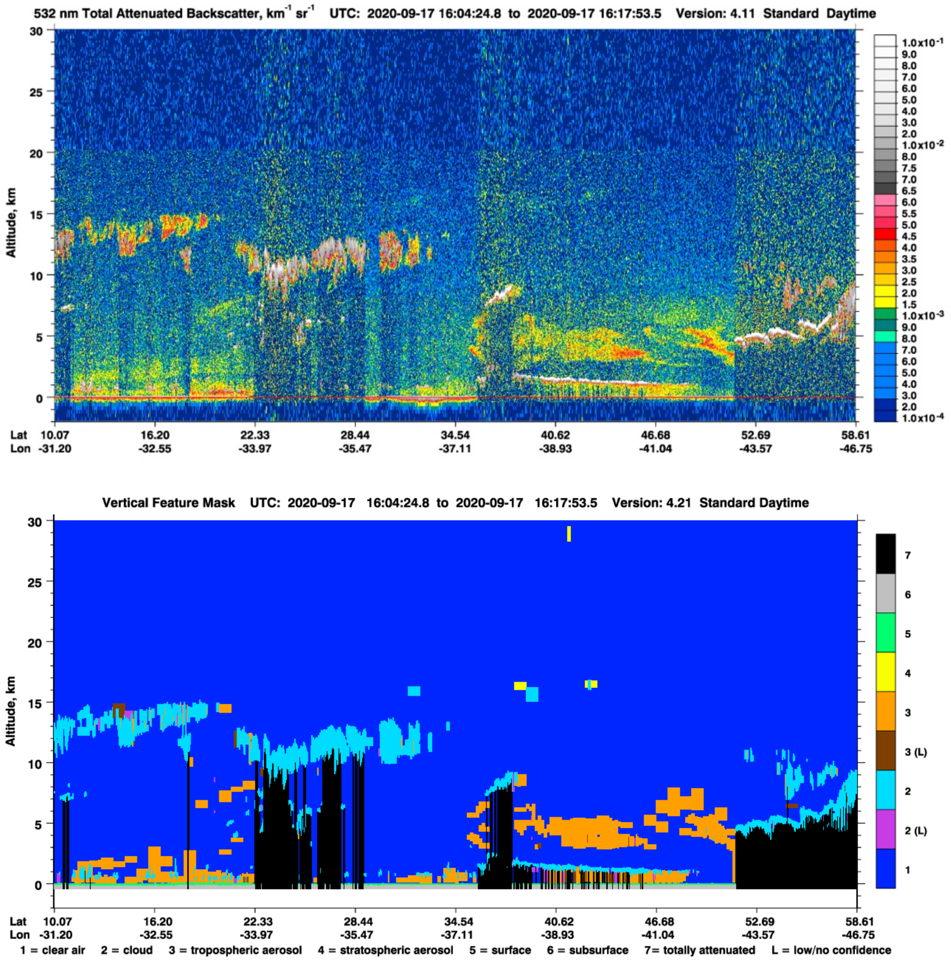


**Supplementary Figure S2.** Same as Figure S1 for the eastern CALIOP curtain used in Fig. 2. Image credits: NASA LaRC https://www-calipso.larc.nasa.gov/.

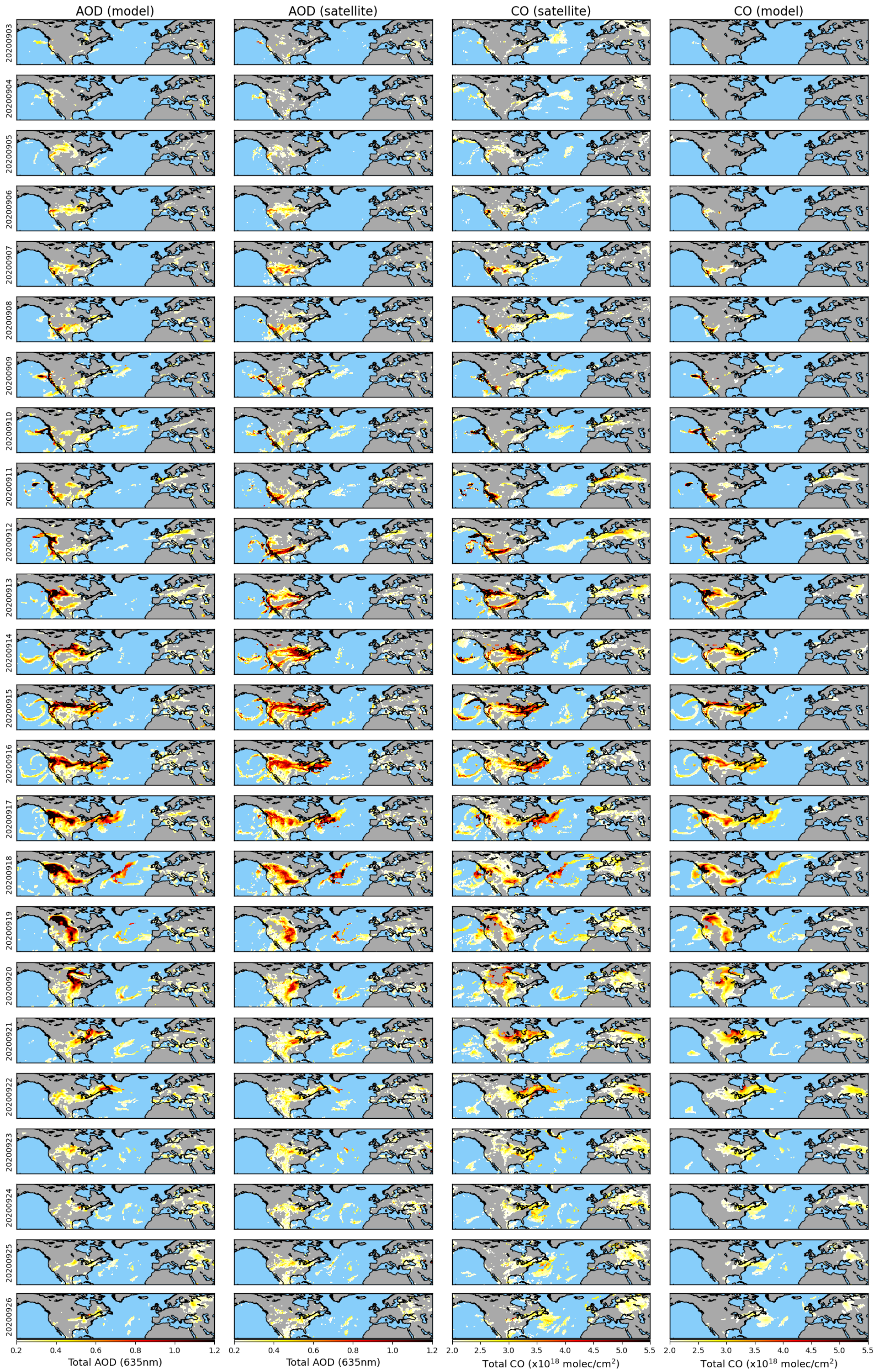


**Supplementary Figure S3.** Daily evolution of CAMS AOD, GEO-ring AOD, IASI CO, and CAMS CO, from 3 to 26 September 2020. Note that AOD and CO are shown for values larger than 0.2 and $2\times10^{18}$ molec/cm$^2$, respectively, to highlight thick plumes only. Regions corresponding to heavy cloud cover and coarse particles were masked in the data preprocessing.
